# Gfi1ab-Cebpβ axis promotes eosinophilopoiesis in zebrafish

**DOI:** 10.1097/BS9.0000000000000314

**Published:** 2026-11-02

**Authors:** Hao Wen, Zihan Hu, Mei Wu, Wan Peng, Yicong Sun, Jing Li, Junwei Lian, Yubing Huang, Yiyue Zhang, Gaofei Li

**Affiliations:** a The Innovation Centre of Ministry of Education for Development and Diseases, Division of Cell, Developmental and Integrative Biology, School of Medicine, South China University of Technology, Guangzhou 510006, China; b Department of Science and Research, School of Medicine, the Sixth Affiliated Hospital of South China University of Technology (Nanhai District People’s Hospital of Foshan), Foshan 528200, China; c Department of Hematology, School of Medicine, The Second Affiliated Hospital of South China University of Technology (Guangzhou First People’s Hospital), Guangzhou 510180, China; d Zhanjiang Key Laboratory of Zebrafish Model for Development and Disease, Affiliated Hospital of Guangdong Medical University, Zhanjiang 524023, China; e Blood Transfusion Department, Guangzhou Eighth People’s Hospital, Guangzhou Medical University, Guangzhou, 510440, China

**Keywords:** Cebpβ, Eosinophil, Gfi1ab, Transcriptional regulation, Zebrafish

## Abstract

Eosinophils play essential roles in parasite defense, allergic responses, and tissue homeostasis, yet the regulatory mechanisms governing their development remain incompletely understood. GFI1 is an important regulator of multiple hematopoietic lineages, but its role in eosinophil development is unclear. Using zebrafish as a model, we demonstrate that Gfi1ab promotes eosinophil development, as *gfi1ab* mutation significantly reduces eosinophil numbers, whereas its overexpression enhances eosinophilopoiesis. This effect is probably eosinophil-specific, as *gfi1ab* mutation does not significantly affect erythrocyte, neutrophil, or macrophage development. Integrative RNA-Seq and Chromatin immunoprecipitation sequencing (ChIP-Seq) analyses identify *cebpb* as a potential downstream target of Gfi1ab. Mechanistically, *gfi1ab* deficiency reduces *cebpb* expression, and *cebpb* overexpression partially rescues the eosinophil defects in *gfi1ab*-deficient larvae. These findings identify a Gfi1ab–Cebpβ regulatory axis that promotes eosinophil development and provide new insight into the transcriptional control of eosinophilopoiesis.

## 1. INTRODUCTION

Eosinophils are multifunctional granulocytes involved in host defense, immune regulation, tissue homeostasis, and pathological inflammation.^1^ Dysregulated eosinophil accumulation is associated with several types of cancer^2^ and a range of airway, gastrointestinal, and skin diseases.^3,4^ Therefore, understanding the molecular mechanisms that govern eosinophil development is essential for understanding eosinophil biology and related diseases. During hematopoiesis, the eosinophil lineage arises from eosinophil lineage-committed progenitors in the mammalian bone marrow.^5^ Several transcription factors, including GATA, Ets, CCAAT enhancer-binding protein (C/EBP) family members, and the ID2-TCF3 axis, have been identified as critical regulators of eosinophil lineage specification and differentiation.^6–11^ However, compared with other myeloid cell lineages, the transcriptional regulatory network controlling eosinophilopoiesis remains incompletely defined.

The Growth Factor Independence 1 (GFI1) family consists of evolutionarily conserved zinc-finger transcription factors with essential roles in hematopoietic development.^12,13^ In mammals, GFI1 regulates hematopoietic stem cell maintenance,^14^ myeloid progenitor proliferation,^15^ B cell lineage fate,^16^ and neutrophil differentiation.^17^ Regarding eosinophilopoiesis, GFI1 can inhibit eosinophil major basic protein (MBP) expression in the eosinophilic cell line HL-60 clone 15.^18^ *Gfi1* mutant mice exhibit significantly reduced numbers of thymic eosinophils, although this effect may be eosinophil-intrinsic or secondary to severe systemic inflammation and increased regulatory T cells.^19^ Thus, whether GFI1 participates in homeostatic eosinophil lineage development remains unclear, and addressing this question could provide new insight into the transcriptional regulation of eosinophilopoiesis.

Zebrafish provide an ideal vertebrate model for studying myeloid cell development and function due to their genetic conservation, external development, optical transparency, and amenability to in vivo genetic manipulation.^20^ A recent study established eosinophil-specific lectin (*eslec*) as a specific marker of zebrafish eosinophils and revealed their spatiotemporal developmental pattern.^21^ Furthermore, a Cebp1-Cebpβ transcriptional axis was reported to regulate eosinophilopoiesis, highlighting the key role of Cebpβ in promoting eosinophil lineage fate in zebrafish.^21^ In the zebrafish genome, *gfi1aa* and *gfi1ab* are 2 orthologs of human *GFI1*, whereas *gfi1b* corresponds to human *GFI1B*.^22,23^ Previous studies have mainly focused on *gfi1aa*, which regulates neutrophil and primitive erythrocyte development.^24–27^ In contrast, the function of *gfi1ab* in hematopoiesis, particularly in myelopoiesis, remains poorly characterized.^24^ Therefore, whether zebrafish Gfi1aa and Gfi1ab contribute to eosinophilopoiesis warrants further investigation.

In the present study, we identify Gfi1ab as a critical positive regulator of eosinophil development in zebrafish. We demonstrate that loss of *gfi1ab* markedly impairs eosinophilopoiesis, whereas enforced *gfi1ab* expression promotes eosinophil development. Mechanistically, integrated transcriptomic and chromatin occupancy analyses identify *cebpb* as a downstream target of Gfi1ab, and functional rescue experiments support a Gfi1ab-Cebpβ regulatory axis that promotes eosinophil lineage development. Together, our findings establish *gfi1ab* as a previously unrecognized regulator of eosinophilopoiesis and expand the transcriptional regulatory network governing eosinophil lineage development.

## 2. MATERIALS AND METHODS

### 2.1. Animals

Zebrafish were maintained and bred according to *The Zebrafish Book*.^28^ The zebrafish lines used in this study were as follows: AB wild-type strain, *gfi1aa*^smu10^ (*gfi1aa*^−/−^ hereafter) mutant,^25^ *gfi1ab*^*smu*12^ (*gfi1ab*^−/−^ hereafter) mutant,^24^ and *Tg(eslec:eGFP*).^21^ All work involving zebrafish was reviewed by the Animal Ethics Committee of South China University of Technology (SCUT).

### 2.2. Whole-mount in situ hybridization

Whole-mount in situ hybridization (WISH) was performed according to a previously described standard protocol.^29^ Larvae were collected at 3, 5, or 7 days post-fertilization (dpf) and stained with probes for *lyz* (neutrophils), *mfap4* (macrophages), *βe-1* (erythrocytes), or *eslec* (eosinophils), which serve as lineage markers for zebrafish.^21,30–32^ WISH signals were manually quantified across individual Z-stack planes and acquired using a stereo fluorescence microscope (Zeiss, Oberkochen, Germany). Images from different planes were subsequently combined using the ZEN software.

### 2.3. Periodic Acid-Schiff staining

Adult *gfi1ab*^*+/+*^ and *gfi1ab*^*−/−*^ zebrafish (3 months old) were anesthetized and euthanized prior to kidney marrow (KM) cell isolation. Collected KM cells were cytospun onto glass slides using a Shandon Cytospin 4 centrifuge (ThermoFisher, Waltham, Massachusetts). The resulting cell smears were stained with a Periodic Acid-Schiff (PAS) staining kit (Solarbio, Beijing, China, G1281) according to the manufacturer’s instructions. Images were acquired using a VS200 microscope (Olympus, Tokyo, Japan) and analyzed with OlyVIA software.

### 2.4. Transient overexpression in zebrafish larvae

pTol2 plasmids containing the *hsp70* promoter upstream of sequences encoding Gfi1ab-eGFP and Cebpβ-eGFP fusion proteins were constructed and individually injected into one-cell-stage zebrafish embryos. Control (Ctrl) embryos were injected with pTol2*-hsp70:eGFP* control plasmids. The injected larvae were heat-shocked daily at 39°C for 2 hours from 3 to 7 dpf to induce overexpression. At 7 dpf, larvae exhibiting strong fluorescence were selected for subsequent WISH analysis.

### 2.5. Reverse transcription quantitative PCR (RT-qPCR)

Total RNA was extracted using TRIzol reagent (ThermoFisher) from sorted eosinophils or from 7 dpf larvae. cDNA was synthesized using the HiScript II Q Select RT SuperMix kit (Vazyme, Nanjing, China). Quantitative PCR was performed on a LightCycler 96 system (Roche, Basel, Switzerland) with SYBR Green PCR reagents (ThermoFisher). Relative expression levels were calculated by the 2^−ΔΔCt^ method using *ef1α* as the internal control. Primer sequences are as follows: *ef1α* (forward primer [FP]: GAGAAGTTCGAGAAGGAAGC; reverse primer [RP]: CGTAGTATTTGCTGGTCTCG), *gfi1ab* (FP: GGTTCACCAGACTCTCACCT; RP: CGAGTACCTGCTCCAAGCGT), and *cebpb* (FP: TGCCCCAGTACCAGCATCTGG; RP: CGCTCGGTCAGCGAGATGTAGT).

### 2.6. Bulk RNA-Seq

To compare transcriptomes of *gfi1ab*^*+/+*^ and *gfi1ab*^*−/−*^ eosinophils, 7 dpf *gfi1ab*^*+/+*^; *Tg(eslec:eGFP*) and *gfi1ab*^*−/−*^; *Tg(eslec:eGFP*) larvae were ground and digested with Dispase (ThermoFisher) to generate single-cell suspensions. Cell suspensions were subjected to fluorescence-activated cell sorting (FACS), and enhanced green fluorescent protein (eGFP)-positive eosinophils were collected for library construction (~100 cells per library). Library preparation was performed based on the Smart-Seq2 protocol,^33^ with reaction volumes increased and amplification cycles induced to accommodate the larger cell input relative to the original single-cell design. Sequencing was performed on NovaSeq 6000 (Illumina, San Diego, California). Reads were aligned to the zebrafish genome (GRCz11) using STAR,^34^ and mapped reads were quantified with featureCounts.^35^ Differentially expressed genes (DEGs) were analyzed using DESeq2.^36^ Gene Ontology (GO)/Kyoto Encyclopedia of Genes and Genomes (KEGG) analysis was performed with Metascape^37^ and visualized with ggplot2.

### 2.7. Chromatin immunoprecipitation sequencing

Chromatin immunoprecipitation sequencing (ChIP-Seq) was performed to identify genomic binding sites of Gfi1ab. Experimental procedures and downstream data analysis were carried out based on the previously reported protocol^25^ with minor modifications. Briefly, zebrafish embryos injected with pTol2*-hsp70:gfi1ab-eGFP* at the one-cell stage were raised to 12 hours post-fertilization (hpf), heat-shocked at 39°C for 2 hours, recovered for 1 hour, and fixed for cross-linking at 15 hpf. Chromatin was sonicated using a Covaris M220 ultrasonicator. Input samples were collected from the sonicated products (1% volume) as controls, and the remaining material was incubated with anti-GFP antibody (1:200, Abcam, Cambridge, UK). Antibody–DNA complexes were enriched using Dynabeads Protein G (ThermoFisher), crosslinks were reversed, and DNA was purified. Libraries were constructed using the NEBnext Ultra II kit (New England Biolabs, Ipswich, Massachusetts) and sequenced on a NovaSeq 6000 platform (Illumina). Reads were aligned with BWA-MEM,^38^ filtered with Samtools,^39^ peaks were called with MACS2,^40^ and annotation was performed with ChIPseeker.^41^

### 2.8. Luciferase assay

To evaluate the regulatory effect of Gfi1ab on the *cebpb* promoter, the *cebpb* promoter fragment was cloned into the pGL4.10 (Promega, Madison, Wisconsin) luciferase reporter vector. The Gfi1ab-overexpression pTol2 plasmid was constructed, and the corresponding empty vector was used as a control. HEK-293T cells were co-transfected with either the *cebpb* promoter-reporter plasmid or the empty pGL4.10 vector, together with either the Gfi1ab-overexpression plasmid or the control vector, and pGL4.75[hRluc/CMV] (Promega) as an internal control. Cells were lysed 48 hours after transfection, and firefly and Renilla luciferase activities were measured using the Dual-Luciferase^®^ Reporter Assay System (Promega).

### 2.9. Statistical analysis

All experimental data were processed and analyzed using GraphPad Prism 10. Statistical significance was determined using Student *t* test for comparisons between 2 normally distributed groups, 2-way analysis of variance (ANOVA) followed by Tukey post hoc test for comparisons among multiple groups, or Fisher exact test for categorical variables where appropriate. Data are presented as the mean ± standard error of the mean (SEM). *p* values < 0.050 were considered statistically significant.

## 3. RESULTS

### 3.1. Gfi1ab promotes eosinophil development in zebrafish

As described earlier, *gfi1aa* and *gfi1ab* are two zebrafish orthologs of the mammalian *GFI1* gene. To determine their respective roles in eosinophil development, we analyzed previously established *gfi1aa* and *gfi1ab* mutant zebrafish lines. WISH showed that *gfi1aa* deficiency did not affect eosinophil numbers compared with sibling controls (**Fig. 1A and B**), indicating that Gfi1aa is dispensable for eosinophil development. In contrast, *gfi1ab* mutant larvae exhibited a marked reduction in *eslec*-positive eosinophils (**Fig. 1C and D**), indicating that Gfi1ab promotes eosinophil development in larval zebrafish. To further determine whether *gfi1ab* is also required for adult eosinophilopoiesis, KM cells from 3-month-old adult zebrafish were subjected to PAS staining. PAS-positive eosinophils were markedly decreased in *gfi1ab* mutants compared with wild-type (WT) controls (**Fig. 1E and F**). Together, these results demonstrate that *gfi1ab*, but not *gfi1aa*, is required for eosinophilopoiesis in both larval and adult zebrafish.

**Figure 1.**
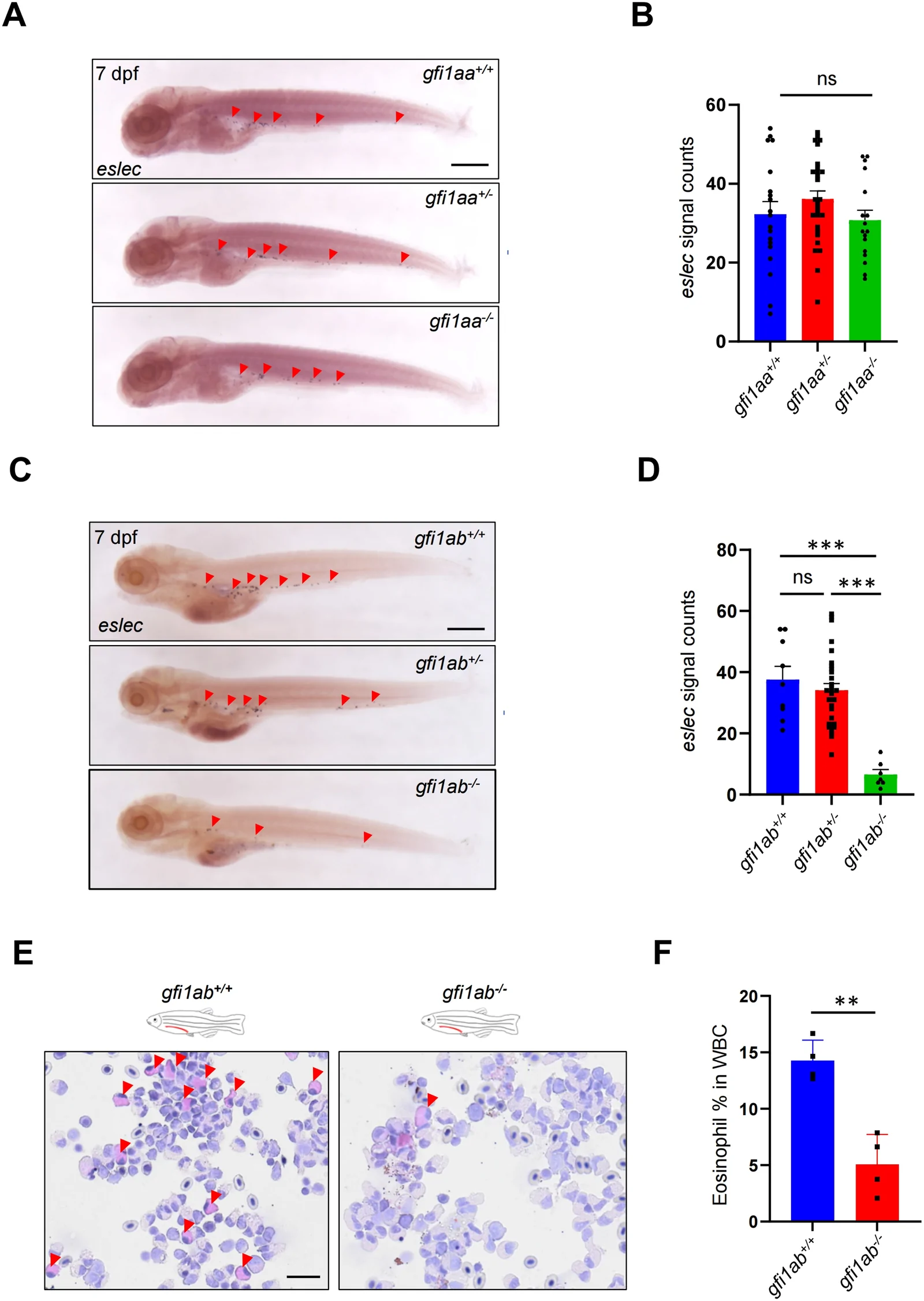
Gfi1ab, but not Gfi1aa, is required for eosinophil development in zebrafish. (A–B) No eosinophil defect was observed upon *gfi1aa* mutation. WISH for *eslec* was performed in 7 dpf *gfi1aa*^+/+^, *gfi1aa*^+/−^, and *gfi1aa*^−/−^ larvae. Red arrowheads indicate eosinophils. Scale bar: 200 μm. Quantification analysis showed no significant difference among the indicated genotypes (mean ± SEM, one-way ANOVA followed by Tukey multiple comparisons test). (C–D) Impaired eosinophilopoiesis upon *gfi1ab* mutation. WISH for *eslec* was performed in 7 dpf *gfi1ab*^+/+^, *gfi1ab*^+/−^, and *gfi1ab*^−/−^ larvae. Red arrowheads indicate eosinophils. Scale bar: 200 μm. Quantification analysis showed significantly reduced numbers of eosinophils in *gfi1ab*^−/−^ larvae compared with controls (mean ± SEM, one-way ANOVA followed by Tukey multiple comparisons test). (E–F) Decreased eosinophils in adult *gfi1ab*^−/−^ zebrafish KM. Red arrowheads indicate PAS-positive eosinophils. Scale bar: 20 μm. Quantification analysis indicated that *gfi1ab* deficiency significantly reduced eosinophil abundance in adult zebrafish (mean ± SEM, unpaired two-tailed *t* test with Welch’s correction). ns, not significant; **p < 0.010; ***p < .001. ANOVA = analysis of variance, PAS = periodic acid-Schiff, SEM = standard error of the mean, WISH = whole-mount in situ hybridization.

To further rule out potential off-target mutations in the *gfi1ab* mutants, we examined whether overexpression of *gfi1ab* can rescue the mutant phenotype. We generated a heat shock-inducible *hsp70:gfi1ab-eGFP* overexpression construct and injected it into zebrafish embryos at the one-cell stage (**Fig. 2A**). Embryos were subjected to daily heat shock from 3 to 7 dpf to induce transgene expression (**Fig. 2A**). RT-qPCR confirmed robust upregulation of *gfi1ab* transcript levels after heat shock induction, indicating successful transgene overexpression (**Fig. 2B**). WISH revealed that enforced *gfi1ab* expression significantly increased eosinophil numbers (**Fig. 2C and D**). Importantly, overexpression of *gfi1ab* in the *gfi1ab*-deficient background markedly restored eosinophil numbers relative to mutant controls (**Fig. 2C and D**), demonstrating substantial rescue of the developmental defect caused by *gfi1ab* loss. Together, these results further validate that Gfi1ab positively regulates eosinophilopoiesis in zebrafish in vivo.

**Figure 2.**
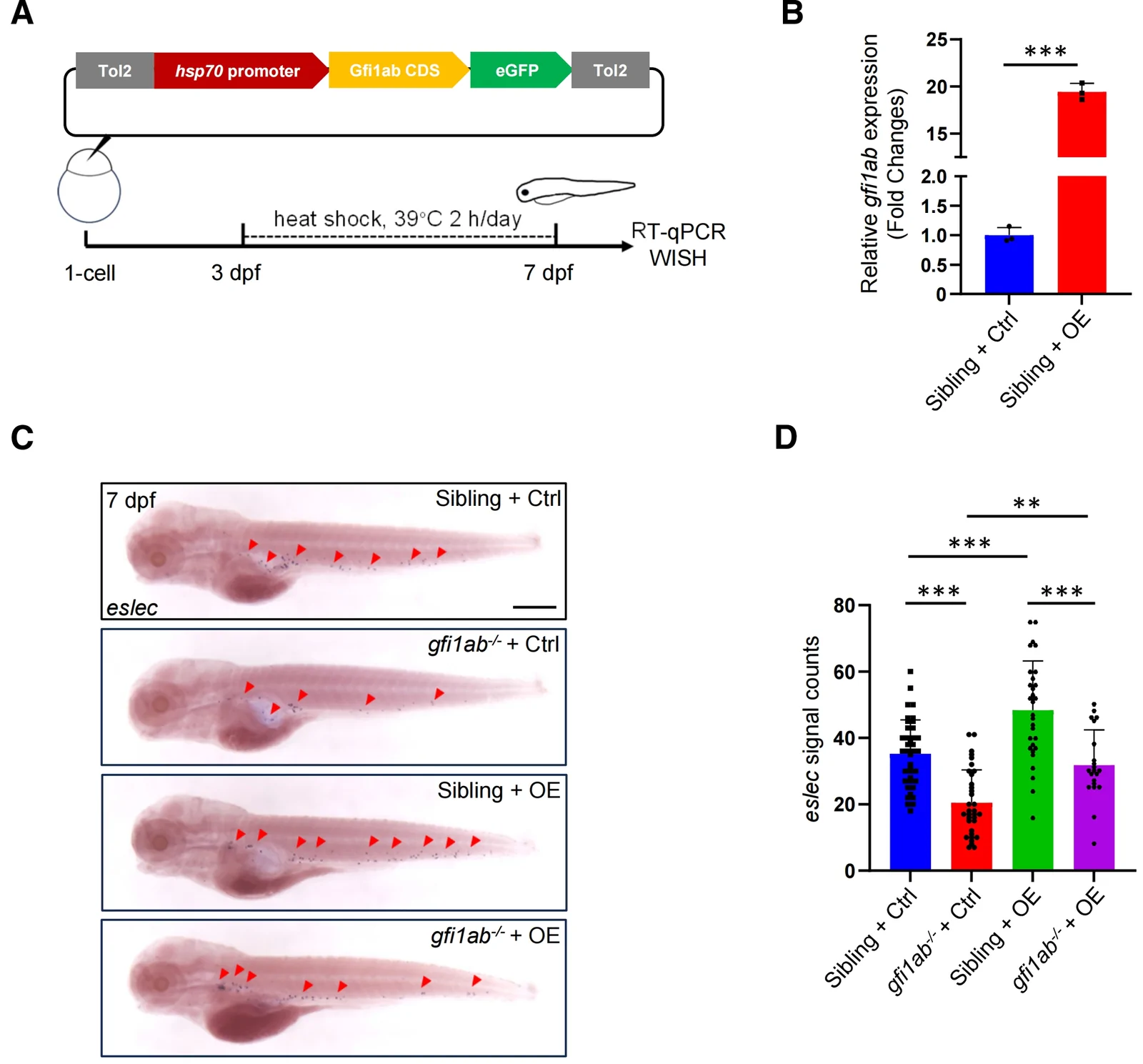
Gfi1ab overexpression promotes eosinophil development and rescues *gfi1ab* deficiency. (A) Schematic diagram of the *hsp70:gfi1ab-eGFP* construct and experimental workflow. Plasmids were injected at the one-cell stage (25 pg per embryo). Heat shock was applied daily at 39°C for 2 h from 3 to 7 dpf to induce Gfi1ab overexpression. (B) RT-qPCR analysis confirming successful *gfi1ab* overexpression. Data were analyzed using an unpaired 2-tailed *t* test with Welch correction (mean ± SEM). (C–D) WISH showing that Gfi1ab overexpression rescued eosinophilopoiesis defects of *gfi1ab* mutants. Ctrl represents larvae injected with pTol2*-hsp70:eGFP* plasmids as a control. Red arrowheads indicate eosinophils. Scale bar: 200 μm. Quantification analysis confirmed that *gfi1ab* deficiency significantly reduced eosinophil numbers, whereas Gfi1ab-overexpression increased eosinophil abundance and partially rescued the eosinophil defect in *gfi1ab* mutants (mean ± SEM, two-way ANOVA). ns, not significant; **p < 0.010; ***p < .001. ANOVA = analysis of variance, CDS = coding sequences, PAS = Periodic Acid-Schiff, RT-qPCR = reverse transcription quantitative PCR, SEM = standard error of the mean, WISH = whole-mount in situ hybridization.

### 3.2. Gfi1ab does not affect neutrophil, macrophage, or erythrocyte lineage

To determine whether the eosinophil defect observed in *gfi1ab* mutants reflects a broader hematopoietic abnormality, we next examined additional hematopoietic lineages during zebrafish development. WISH was performed at 3 dpf using *lyz* and *mfap4* as markers for neutrophils and macrophages, respectively. We found that *gfi1ab* deficiency did not significantly alter the numbers of *lyz*-positive neutrophils or *mfap4*-positive macrophages compared with sibling controls (**Fig. 3A and D**), demonstrating that myeloid lineages other than eosinophils are largely unaffected by loss of *gfi1ab*.

**Figure 3.**
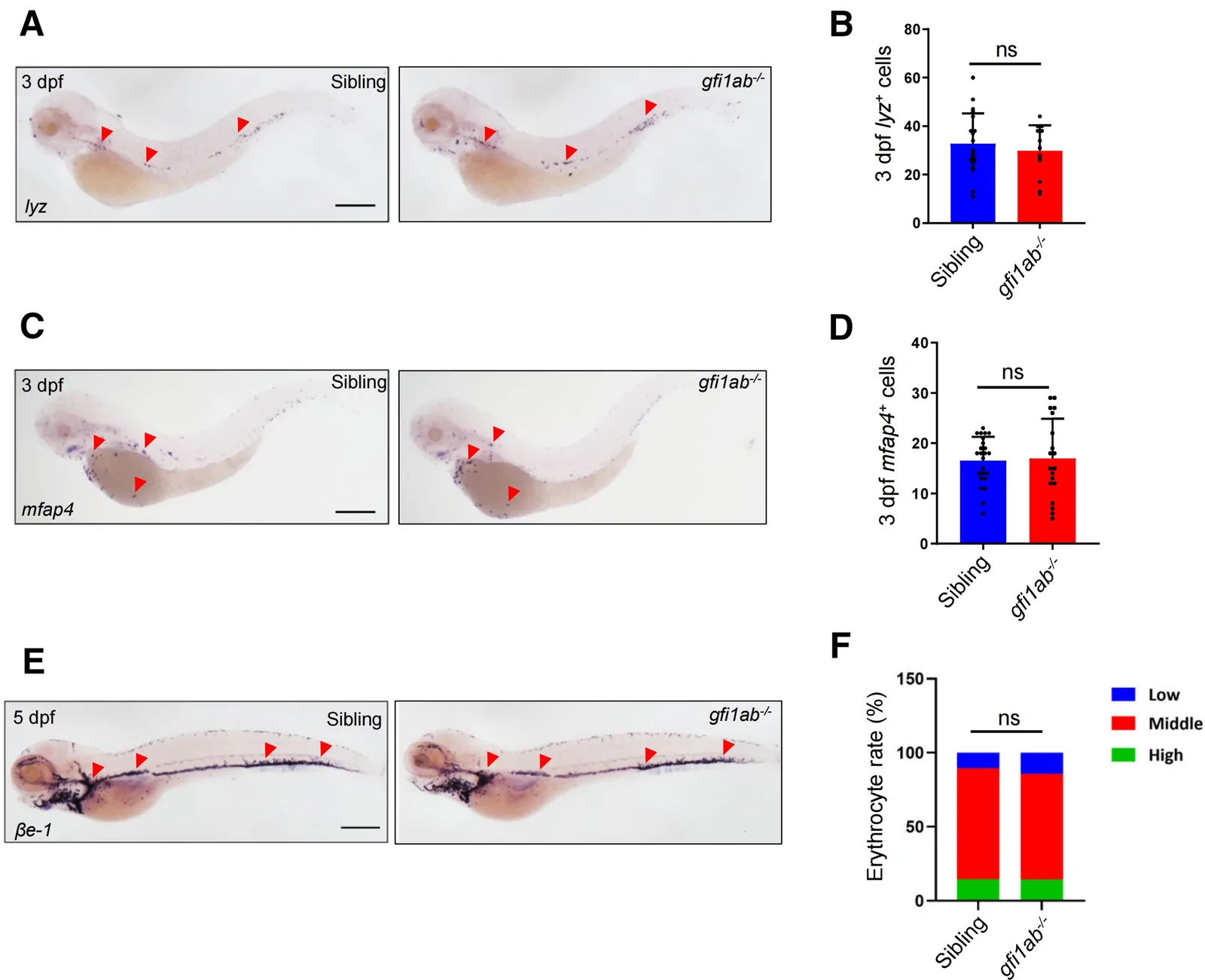
*gfi1ab* deficiency does not significantly affect other hematopoietic lineages. (A–B) WISH showing no neutrophil defect in *gfi1ab*-deficient larvae. Red arrowheads indicate *lyz*^+^ neutrophils. Scale bar: 200 μm. Quantification analysis showed no significant difference between sibling controls and mutants (mean ± SEM, unpaired 2-tailed *t* test with Welch correction). (C–D) WISH showing no macrophage defect in *gfi1ab*-deficient larvae. Red arrowheads indicate *mfap4*^+^ macrophages. Scale bar: 200 μm. Quantification analysis showed no significant difference between sibling controls and mutants (mean ± SEM, unpaired 2-tailed *t* test with Welch correction). (E–F) WISH showing no erythrocyte defect in *gfi1ab*-deficient larvae. Red arrowheads indicate *βe-1*^+^ erythrocytes. Scale bar: 200 μm. Erythroid phenotypes were categorized as low, medium, or high. Statistical analysis was performed using Fisher exact test. ns, not significant. SEM = standard error of the mean, WISH = whole-mount in situ hybridization.

To assess whether erythropoiesis is affected, we analyzed the expression of the erythroid marker *βe-1* in 5 dpf larvae. Based on staining intensity, larvae were categorized into low, medium, or high intensity groups. No significant difference in erythroid distribution was observed between *gfi1ab* mutants and siblings (**Fig. 3E and F**), indicating that *gfi1ab* deficiency does not affect erythroid development.

Together, these data suggest that the hematopoietic defects caused by *gfi1ab* deficiency are not broadly distributed across major hematopoietic lineages and instead indicate a specific requirement for *gfi1ab* in eosinophilopoiesis.

### 3.3. Gfi1ab activates *cebpb* transcription

To identify candidate genes directly targeted by Gfi1ab, we performed mini-bulk RNA-Seq and ChIP-Seq. RNA-Seq identified DEGs affected by *gfi1ab* mutation, while ChIP-Seq identified genes associated with Gfi1ab binding (**Fig. 4A**). Based on the sample-to-sample distance heatmap (**Fig. 4B**), intergroup differences were greater than intragroup differences. Integrated analysis revealed a subset of downregulated genes in mutants showing direct Gfi1ab binding (**Fig. 4A**), suggesting that these genes may be directly activated by Gfi1ab. GO/KEGG annotation suggested that these genes are associated with pathways such as protein processing in the endoplasmic reticulum (ER) and protein transport (**Fig. 4C**). In addition, among the candidate genes, *cebpb* attracted particular attention because of its strong Gfi1ab-binding signal and marked downregulation in *gfi1ab* mutants (**Fig. 4D and E**). Given the established role of *cebpb* in promoting eosinophil cell fate,^21^ we hypothesized that *cebpb* may function as a downstream effector of Gfi1ab during eosinophil development.

**Figure 4.**
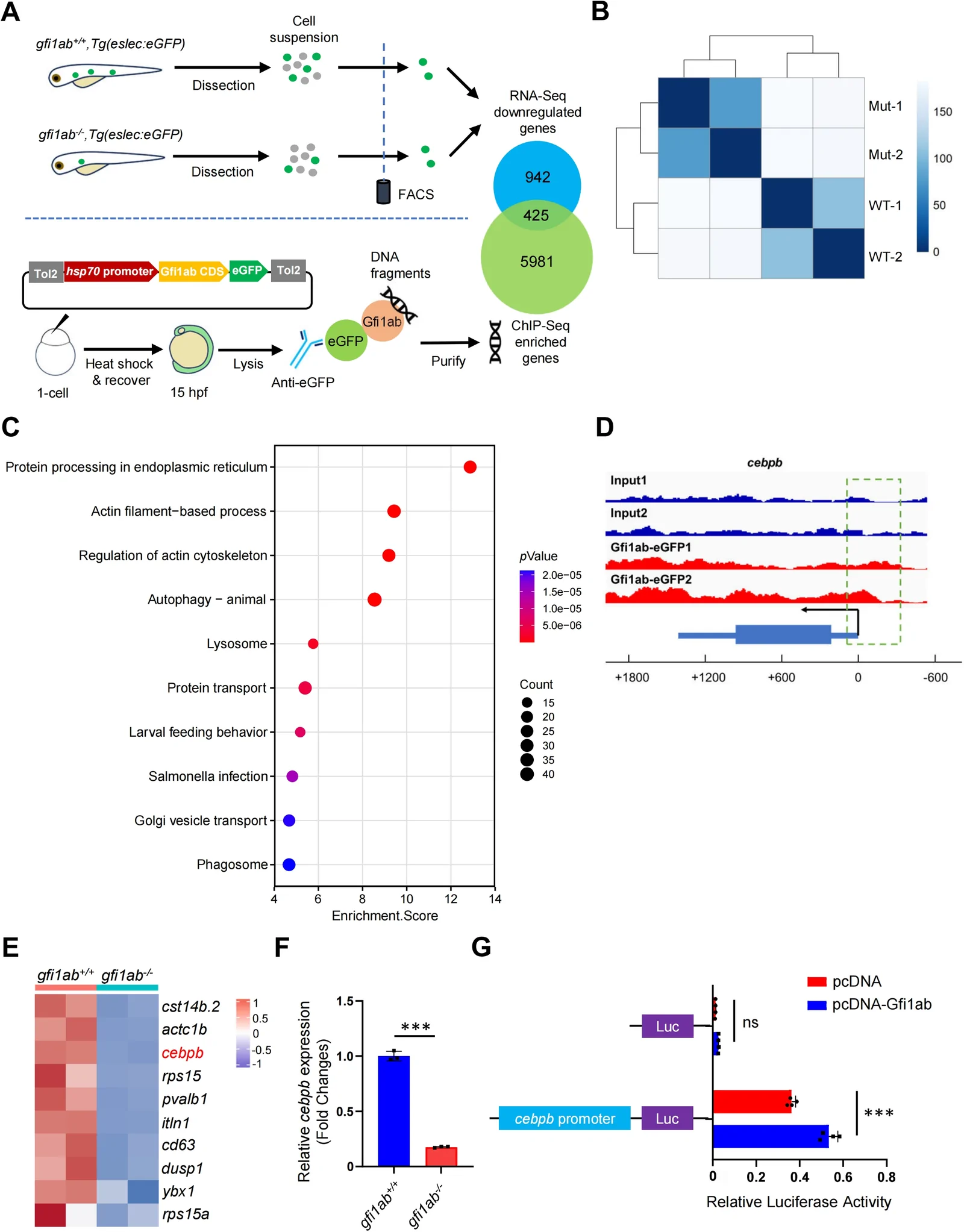
Gfi1ab activates *cebpb* transcription. (A) Schematic overview of the experimental strategy. Bulk RNA-seq was performed on eosinophils isolated from WT and mutant larvae to identify DEGs upon *gfi1ab* mutation (*q* < 0.05, baseMean > 50, |log_2_ fold change| > 1). One-cell-stage embryos were injected with *hsp70:gfi1ab-eGFP* constructs, heat-shocked to induce Gfi1ab overexpression, and lysed for ChIP-seq to determine genomic binding targets of Gfi1ab (*q* < 0.001, TSS ± 2 kb). Overlap analysis of the RNA-Seq and ChIP-Seq datasets identified candidate target genes that were downregulated in *gfi1ab* mutants and associated with Gfi1ab-binding peaks. (B) Sample-to-sample heatmap of the RNA-Seq samples. Mut represents *gfi1ab* mutants. (C) GO/KEGG analysis of candidate genes. (D) ChIP-Seq tracks showing Gfi1ab-binding peaks at the *cebpb* promoter (boxed). (E) Heatmap showing expression changes of the top 10 overlapping candidate genes (ranked by baseMean), highlighting *cebpb* as a markedly downregulated gene in *gfi1ab* mutants. (F) RT-qPCR validation of *cebpb* expression in sorted eosinophils. *gfi1ab* deficiency significantly reduced *cebpb* expression (mean ± SEM, 2-tailed unpaired *t* test with Welch correction). (G) Luciferase reporter assay showing that Gfi1ab overexpression significantly enhanced *cebpb* promoter activity. Data are presented as mean ± SEM. Statistical significance was determined using one-way ANOVA followed by Tukey multiple comparisons test. ns, not significant; ***p < .001. ANOVA = analysis of variance, ChIP-seq = chromatin immunoprecipitation sequencing, DEG = differentially expressed gene, GO/KEGG = Gene Ontology/Kyoto Encyclopedia of Genes and Genomes, RT-qPCR = reverse transcription quantitative PCR, SEM = standard error of the mean, TSS = transcription start site, WT = wild-type.

To validate the transcriptional regulation of *cebpb* by Gfi1ab, eosinophils were isolated from *gfi1ab* mutant zebrafish and subjected to RT-qPCR analysis. We found that *cebpb* expression was significantly reduced in *gfi1ab*-deficient eosinophils compared with WT controls (**Fig. 4F**), further supporting the positive regulation of *cebpb* by Gfi1ab in vivo. To determine whether Gfi1ab directly regulates *cebpb* transcription, we constructed a *cebpb* promoter-driven luciferase reporter and performed dual-luciferase assays. Overexpression of Gfi1ab significantly enhanced *cebpb* promoter activity (**Fig. 4G**), indicating that Gfi1ab directly activates *cebpb* transcription through its promoter region. Together, these results identify *cebpb* as a direct downstream target of Gfi1ab during zebrafish eosinophilopoiesis.

### 3.4. Gfi1ab-Cebpβ axis promotes eosinophil development

To further determine whether Cebpβ functions downstream of Gfi1ab during eosinophil development, we generated a heat shock-inducible pTol2*-hsp70:cebpb-eGFP* overexpression construct and injected it into zebrafish embryos. After daily heat shock from 3 to 7 dpf, RT-qPCR confirmed robust upregulation of *cebpb* expression in the larvae, indicating successful transgene expression (**Fig. 5A and B**). WISH further showed that *cebpb* overexpression significantly increased eosinophil numbers in siblings and *gfi1ab* mutants, partially restoring the eosinophil defect caused by *gfi1ab* loss (**Fig. 5C and D**). These results indicate that Cebpβ promotes eosinophilopoiesis and contributes to the pro-eosinophilic function of Gfi1ab, supporting a Gfi1ab-Cebpβ regulatory axis that promotes eosinophil development in zebrafish.

**Figure 5.**
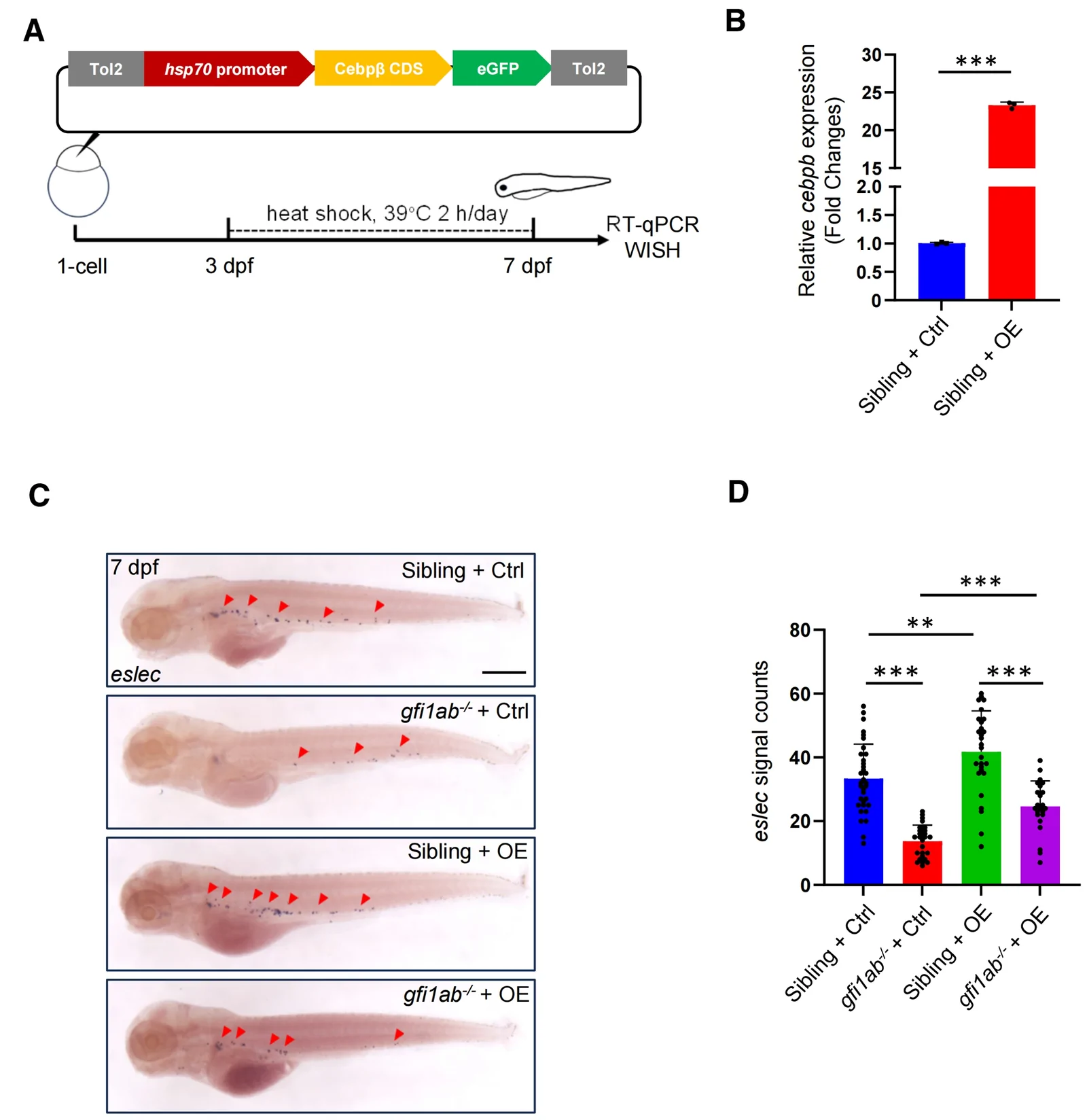
Cebpβ promotes eosinophil development downstream of Gfi1ab. (A) Schematic diagram of the *hsp70:cebpb-eGFP* construct and experimental workflow. Plasmids were injected at the one-cell stage (25 pg per embryo). Heat shock was applied daily at 39°C for 2 h from 3 to 7 dpf to induce Cebpβ overexpression. (B) RT-qPCR analysis confirming successful *cebpb* overexpression (mean ± SEM, unpaired 2-tailed *t* test with Welch correction). (C–D) WISH showing that Cebpβ-overexpression rescued eosinophilopoiesis defects of *gfi1ab* mutants. Ctrl represents larvae injected with pTol2*-hsp70:eGFP* plasmids as a control. Red arrowheads indicate eosinophils. Scale bar: 200 μm. Quantification analysis indicated that Cebpβ over-expression increased eosinophil abundance and partially rescued the eosinophil defect in *gfi1ab* mutants (mean ± SEM, two-way ANOVA followed by Tukey multiple comparisons test). **p < 0.010; ***p < .001. ANOVA = analysis of variance, SEM = standard error of the mean, WISH = whole-mount in situ hybridization.

## 4. DISCUSSION

In this study, we identify *gfi1ab* as a previously unrecognized regulator of eosinophilopoiesis in zebrafish. Loss of *gfi1ab* specifically impairs eosinophil development without affecting neutrophil, macrophage, or erythrocyte lineages, whereas enforced *gfi1ab* expression promotes eosinophil production and rescues the mutant phenotype. Mechanistically, our data support a model in which Gfi1ab promotes eosinophil development through transcriptional activation of *cebpb*, thereby establishing a Gfi1ab-Cebpβ axis that drives eosinophilopoiesis.

Although several transcription factors have been implicated in eosinophilopoiesis, new regulators controlling eosinophil lineage development remain to be discovered. GFI1 can inhibit MBP expression in HL-60 clone 15 cells, while its precise role in eosinophilopoiesis remains unclear.^18^ *Gfi1* mutant mice have significantly fewer thymic eosinophils, though they also exhibit increased regulatory T cells that may suppress eosinophil responses during systemic inflammation.^19^ Therefore, whether mammalian GFI1 specifically regulates eosinophilopoiesis under homeostatic conditions remains unclear. Our findings identify Gfi1ab as a novel positive regulator of zebrafish eosinophil development. In addition, our data indicate that *gfi1ab* mutation does not affect erythrocyte, neutrophil, and macrophage development, further suggesting a specific role for Gfi1ab in controlling eosinophil development.

Another finding of this study is the apparent functional divergence between the two zebrafish *GFI1* orthologues, *gfi1aa* and *gfi1ab*. Previous studies have shown that Gfi1aa regulates neutrophil and primitive erythrocyte development,^24–27^ whereas our data indicate that Gfi1aa is dispensable for eosinophil development and that Gfi1ab is specifically required. This divergence likely reflects subfunctionalization following teleost genome duplication.^42^ The broader hematopoietic abnormalities observed following mammalian *GFI1* deficiency,^14–17^ further support the possibility of paralogue partitioning in zebrafish hematopoiesis.

Although GFI1 proteins are classically characterized as transcriptional repressors through recruitment of chromatin-modifying co-repressor complexes,^43^ increasing evidence suggests that their regulatory activities are context-dependent and may extend beyond canonical transcriptional repression. For instance, GFI1 upregulates *RASGRP2* expression in lung cancer cells.^44^ In group 2 innate lymphoid cells, aryl hydrocarbon receptor (AHR) cooperates with GFI1 to promote *Ahr* expression.^45^ In neutrophils, GFI1 cooperates with C/EBPε to upregulate the expression of neutrophil collagenase.^46^ Our finding that Gfi1ab positively regulates *cebpb* transcription is therefore consistent with the emerging view that GFI1 possesses broader regulatory versatility than previously appreciated. Whether zebrafish Gfi1ab requires cooperation with other transcriptional regulators to activate *cebpb* remains to be determined.

Mechanistically, our data identify Cebpβ as a downstream effector of Gfi1ab during eosinophilopoiesis. Previous work established that Cebpβ, directly inhibited by Cebp1, is a critical positive regulator of eosinophil lineage commitment and probably differentiation in zebrafish.^21^ In the present study, we identified Gfi1ab as a direct activator of *cebpb* expression. Therefore, Cebp1 and Gfi1ab may function antagonistically to regulate *cebpb*, thereby influencing eosinophil lineage fate and differentiation. Whether a mammalian GFI1-C/EBPβ axis similarly regulates eosinophilopoiesis warrants further investigation.

Cebpβ overexpression only partially rescued the eosinophil defect caused by *gfi1ab* deficiency, suggesting that Cebpβ is unlikely to be the sole downstream mediator of Gfi1ab function. Based on GO/KEGG analysis, our data suggest that Gfi1ab may also activate a series of genes related to protein processing in the ER and protein transport. In our previous study, we found that ER stress-related pathways are enriched in eosinophil precursors,^21^ which may reflect granule protein synthesis during eosinophil lineage differentiation. Therefore, Gfi1ab may also directly regulate both *cebpb* and ER-related genes to contribute to eosinophil lineage differentiation.

A limitation of this study is that the cell types and developmental stages used for ChIP-Seq did not fully match those for RNA-seq. Due to the rarity of eosinophils in zebrafish larvae and the high cell input required for ChIP-Seq, we used whole-larval cells for ChIP-Seq, which inevitably introduces background signals from non-eosinophil cells. To address this, we integrated ChIP-Seq with eosinophil-specific RNA-seq to identify Gfi1ab downstream targets in eosinophils. However, differences in developmental stages and the low proportion of eosinophils in the ChIP-Seq samples may have limited our ability to identify Gfi1ab targets in eosinophils. Consistent with this limitation, *cebpb* is unlikely to be the sole downstream target of Gfi1ab, as *cebpb* overexpression only partially rescues the eosinophil defects in *gfi1ab* mutants. Gfi1ab may regulate eosinophilopoiesis through a broader target network, which warrants further investigation.

In summary, our study identifies Gfi1ab as a novel regulator of zebrafish eosinophilopoiesis and demonstrates that Gfi1ab promotes eosinophil development through activation of *cebpb*. These findings provide new evidence that GFI1 participates in eosinophil lineage regulation and further expand the transcriptional network governing eosinophilopoiesis.

## ACKNOWLEDGMENTS

This work was supported by the National Key R&D Program of China (2023YFA1800100), the Guangdong Basic and Applied Basic Research Foundation (2024B1515040019 and 2026A1515012117), and the Medical Scientific Research Foundation of Guangdong Province of China (20251125171844400).

We thank Prof. Jianbin Wang for the methodology of mini-bulk RNA-Seq. We also thank all staff at the research platform and zebrafish facility of SCUT.

## AUTHOR CONTRIBUTIONS

Y.Z. and G.L. contributed to conceptualization, funding acquisition, project administration, resources, supervision, and writing- review & editing. H.W. and G.L. contributed to data curation. H.W. and M.W. contributed to formal analysis. H.W., Z.H., M.W., Y.S., W.P., J.Li., J.Lian, and Y.H. contributed to investigation. H.W., M.W., and G.L. contributed to methodology. H.W. contributed to software and writing-original draft. H.W. and Z.H. contributed to validation. H.W., Z.H., and M.W. contributed to visualization.
